# Peripheral Etanercept Administration Normalizes Behavior, Hippocampal Neurogenesis, and Hippocampal Reelin and GABA_A_ Receptor Expression in a Preclinical Model of Depression

**DOI:** 10.3389/fphar.2018.00121

**Published:** 2018-02-20

**Authors:** Kyle J. Brymer, Erin Y. Fenton, Lisa E. Kalynchuk, Hector J. Caruncho

**Affiliations:** ^1^Department of Psychology, University of Saskatchewan, Saskatoon, SK, Canada; ^2^Division of Medical Sciences, University of Victoria, Victoria, BC, Canada

**Keywords:** etanercept, neurogenesis, reelin, hippocampus, depression, antidepressant

## Abstract

Depression is a serious psychiatric disorder frequently comorbid with autoimmune disorders. Previous work in our lab has demonstrated that repeated corticosterone (CORT) injections in rats reliably increase depressive-like behavior, impair hippocampal-dependent memory, reduce the number and complexity of adult-generated neurons in the dentate gyrus, decrease hippocampal reelin expression, and alter markers of GABAergic function. We hypothesized that peripheral injections of the TNF-α inhibitor etanercept could exert antidepressant effects through a restoration of many of these neurobiological changes. To test this hypothesis, we examined the effect of repeated CORT injections and concurrent injections of etanercept on measures of object-location and object-in-place memory, forced-swim test behavior, hippocampal neurogenesis, and reelin and GABA β2/3 immunohistochemistry. CORT increased immobility behavior in the forced swim test and impaired both object-location and object-in-place memory, and these effects were reversed by etanercept. CORT also decreased both the number and complexity of adult-generated neurons, but etanercept restored these measures back to control levels. Finally, CORT decreased the number of reelin and GABA β2/3-ir cells within the subgranular zone of the dentate gyrus, and etanercept restored these to control levels. These novel results demonstrate that peripheral etanercept has antidepressant effects that are accompanied by a restoration of cognitive function, hippocampal neurogenesis, and GABAergic plasticity, and suggest that a normalization of reelin expression in the dentate gyrus could be a key component underlying these novel antidepressant effects.

## Introduction

There is a strong connection between the immune system and the brain in the context of major depression. Chronic stress induces an inflammatory response, which in the central nervous system includes an increase in pro-inflammatory and a decrease in anti-inflammatory cytokines, in addition to the recruitment of microglia (Dantzer et al., 2008). Exposure to chronic stress is a significant risk factor for the development of depression. One of the earliest links between stress and depression was the observation that stressful life events frequently precede the onset of depression in human populations (reviewed in Sterner and Kalynchuk, 2010). Half of depressed patients display the hallmarks of a deregulated stress response, hypercortisolemia (Sachar and Baron, 1979). Moreover, these high circulating levels of cortisol have pathological consequences for healthy brain functioning (Kendler and Gardner, 2010). Studies of post-mortem tissue from patients with depression have revealed dendritic atrophy in the hippocampus and prefrontal cortex, in addition to reductions in hippocampal volume (Campbell et al., 2004; Banasr et al., 2011). Research using preclinical animal models of depression further corroborate the link between stress and depression. For example, stress significantly increases depressive-like and anhedonia behavior (Sterner and Kalynchuk, 2010). Stress also produces similar neurobiological alterations as those seen in humans, along with decreases in hippocampal cell proliferation, survival, and the rate of neuronal maturation (Lussier et al., 2009, 2013a). Clinically, patients with autoimmune disorders frequently present with comorbid depression (Andersson et al., 2015) – for example, rheumatoid arthritis patients have a lifetime prevalence rate of 13–42% of developing depression (Margaretten et al., 2011) – and there seems to be a clear bidirectional relationship between depression and autoimmune disorders (Euesden et al., 2017). Moreover, subsets of depressed patients exhibit the cardinal features of an inflammatory response, most notably an increased expression of pro-inflammatory cytokines in blood and cerebrospinal fluid (Miller et al., 2009). Some of the most frequently reported elevated pro-inflammatory cytokines include IL-1, IL-6, and TNF-α (Dowlati et al., 2010), and general alterations in neuroimmune systems represent a key component underlying the neurobiology of both mood and psychotic disorders (Calcia et al., 2016; Wohleb et al., 2016; Rodrigues-Amorim et al., 2017).

TNF-α is a soluble cytokine with two receptors, the TNF-α receptor 1 and TNF-α receptor 2. Produced peripherally, TNF-α also acts centrally through activation of toll-like receptor 4 on circumventricular organs and choroid plexus, which in turn activates microglia that then release TNF-α within the brain, with the hippocampus being a key region of expression (McCoy and Tansey, 2008). Within the nervous system, TNF-α has a variety of functions, including modulating cell recruitment and proliferation, and cell death. Under conditions of stress, TNF-α participates in promoting inflammation through stimulating the production of other pro-inflammatory cytokines, including IL-1 and IL-6. Elevated levels of TNF-α produce a wide range of deleterious effects on physiology, emotional behavior, and cognition (Bortolato et al., 2015). TNF-α also increases excitatory synaptic strength by promoting AMPA receptor expression, while concomitantly decreasing inhibitory synaptic strength by inducing internalization of GABA_A_ receptors (Stellwagen et al., 2005). Work in preclinical animal models demonstrates that peripheral administration of TNF-α produces deficits in learning and memory (particularly spatial memory) in addition to anhedonia (Dunn et al., 2005; van Heesch et al., 2013). Furthermore, TNF-α knockout mice display an antidepressant-like phenotype (Yamanda et al., 2000). In an elegant study, Klaus et al. (2016) injected TNF-α peripherally or achieved expression in the hippocampus using viral vectors. Peripheral TNF-α produced anhedonia behavior and increased fear memory, while brain expression increased anxious behavior. Increased expression of TNF-α results in a decrease in neurogenesis through apoptotic neuronal death, whereas low levels of TNF-α promote neurogenesis through increased proliferation of neural precursor cells (Fan et al., 2011). Finally, peripheral administration of the TNF-α inhibitor etanercept decreases depression-like behavior in rats subjected to stress paradigms (Bayramgurler et al., 2013; Krugel et al., 2013; Sahin et al., 2015). In fact, a recent systematic review and meta-analysis validated the effect of anti –TNF-α drugs in improving depression symptoms, and associated the antidepressant effect with baseline symptom severity (Kappelmann et al., 2016).

In spite of the clinical studies showing antidepressant actions of etanercept, little is known about the mechanisms by which anti –TNF-α drugs exert their antidepressant effects. Thereby, considering that administration of peripheral TNF-α produces anhedonia behavior and deficits in spatial memory, we were interested in examining if TNF-α inhibition could alleviate stress-induced deficits in depressive-like behavior and measures of cognition.

Work in our lab has demonstrated that prolonged exposure to stress hormones decreases reelin expressing cells in the dentate subgranular zone, and treatment with the tricyclic antidepressant imipramine reverses this effect (Lussier et al., 2009; Fenton et al., 2015). Reelin is a large extracellular matrix protein primarily expressed by cortical and hippocampal subpopulations of GABAergic interneurons and by cerebellar granule cells (Pesold et al., 1998; Rogers and Weeber, 2008). Within the hippocampus, reelin is involved in a variety of functions, including dendritic spine development and maturation (Niu et al., 2008; Chameau et al., 2009; Hethorn et al., 2015), hippocampal neurogenesis (Teixeira et al., 2011), promoting and modulating maturation and synaptic plasticity of newborn granule cells (Bosch et al., 2016a), and learning and memory (Beffert et al., 2005). Importantly, there is also evidence of decreased reelin expression in the dentate gyrus in postmortem samples from patients with depression (Fatemi et al., 2000). Interestingly, in a mouse model of aging, a viral-induced infection increased inflammation and reduced the number of reelin expressing interneurons (Doehner and Knuesel, 2010). This suggests that the pattern of behaviors (e.g., depression-like behavior, impaired cognition) observed under times of increased inflammatory signaling in chronic stress might be related to a loss of hippocampal reelin expression (Caruncho et al., 2016). Therefore, we hypothesized that reducing chronic stress-induced increases in TNF-α would increase reelin expression and normalize behavior and neurobiological markers. To test this hypothesis, we utilized a repeated corticosterone (CORT) injection model of depression that we and others have shown can produce a robust depressive phenotype (Sterner and Kalynchuk, 2010). Rats received 21 days of CORT or vehicle injections and half the rats in each group also received semi-weekly injections of the TNF-α inhibitor etanercept (Inglis et al., 2005). We then assessed depression-like behavior using the forced-swim test (FST), cognition using the novel object-location and object-in-place tests, and alterations in hippocampal neurogenesis, reelin, and GABA_A_ receptors using immunohistochemistry. Our results support our hypothesis and suggest that peripheral administration of a TNF-α antagonist increases hippocampal reelin expression and corresponding neurobiological markers, and normalizes the behavioral phenotype.

## Materials and Methods

### Animals

We used 24 male adult Long-Evans rats purchased from Charles River (QC, Canada). The rats were reared in standard conditions by the breeder and weighed 200–250 g at the time of arrival in our lab. Rats were 9 weeks old at the start of the experiment. It is generally accepted that at 9 weeks old rats are in the young adult to adulthood stage (Sengupta, 2013). Rats were individually housed in rectangular polypropylene cages containing standard laboratory bedding with access to food and water *ad libitum.* The rodent colony room was maintained at an ambient temperature of 20 ± 1°C on a 12:12 light-dark cycle (lights on at 7 am). All experimental procedures were in accordance with the guidelines of the Canadian Council and Animal Care and an animal care protocol approved by the University of Saskatchewan Committee on Animal Care and Supply. All efforts were made to minimize the number of rats used in the present study.

### Experimental Procedures

**FIGURE 1:**
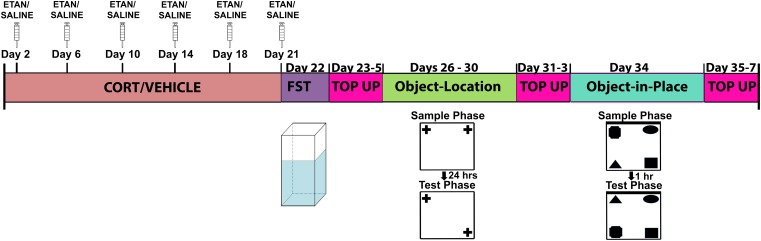
Schematic representation of the experimental design used for the study. All rats received the same amount of handling, injections, and behavioral testing. The injection top up periods in between each behavioral test were implemented to ensure that the effects of CORT did not wane over time.

Rat were handled briefly once per day for 7 days prior to the start of experimental manipulations. We weight-matched the rats and randomly assigned them to one of the following four treatment groups: CORT + saline (*n* = 6), CORT + etanercept (*n* = 6), vehicle + saline (*n* = 6), or vehicle + etanercept (*n* = 6) injections. All CORT and vehicle injections were administered subcutaneously once per day (between 9:00 and 10:00 am) for 21 consecutive days. Etanercept and saline injections were given subcutaneously twice per week (between 2:00 and 3:00 pm) during the 21-day CORT/vehicle injection period. Rats also received 3 days of CORT or vehicle injections and 1 day of etanercept or saline injections in between each behavioral test (forced-swim test, object-location, object-in-place) to maintain the effects of CORT throughout the behavioral testing period. The experimental design is shown in **Figure 1**.

CORT (Steraloids, Newport, RI, United States) was suspended in 0.9% (w/v) physiological saline with 2% (v/v) Tween-80 (Sigma–Aldrich) and given at a dose of 40 mg/kg at a volume of 1 ml/kg. This dose produces reliable and robust increases in depression-like behavior (Gregus et al., 2005; Johnson et al., 2006). Etanercept (Immunex, Thousand Oaks, CA, United States) was suspended in 0.9% (w/v) physiological saline with 2% (v/v) Tween-80 and given at a dose of 0.8 mg/kg in a volume of 1 ml/kg. This dose was chosen based on previous studies showing that etanercept can normalize depression-like behavior in rats (Bayramgurler et al., 2013).

Body weight was recorded for each rat during the injection phase of the experiment.

### Behavioral Testing

Behavioral testing was conducted in a procedures room that was not used for any other aspect of the study. All behaviors were recorded with a digital video camera and stored for offline analyses by researchers blind to the treatment conditions. The FST assessed depression-like behavior, whereas the novel object location and object-in-place tests assessed recognition memory.

### Forced Swim Test

The FST was conducted the day after the final CORT injection. We used a modified version of the Porsolt test, as previously described (Marks et al., 2009). Each rat was individually placed in a Plexiglas swim tank (25 cm diameter × 60 cm high, 27 ± 2°C water, 30 cm deep) for 10 min. We measured the duration of time each rat spent immobile, struggling, and swimming.

### Object Recognition Memory

Recognition memory was assessed according to previously published protocols (Howland and Cazakoff, 2010; Howland et al., 2012). Object-location (OBL) memory testing took place in a square open-field area (60 × 60 × 60 cm) constructed of white corrugated plastic. Rats received three habituation sessions before the training session. During the first two habituation sessions, rats were brought in pairs and placed in separate arenas for 10 min. During the last habituation session, rats were brought in individually and placed in the arena for 10 min. Rats were immediately returned to the colony room after each habituation session. The last habitation session occurred 24–48 h. before the first testing session. The objects used in this test were made of porcelain and did not exceed 10 cm in height or length. Duplicate copies of objects were used when the same objects were required for both training and testing phases. Object locations were counterbalanced to eliminate potential side preferences in all tests. In both sample and test phases, objects were placed in the corners of the arena 10 cm from each of the nearest walls and rats were placed in the center of the arena facing the wall opposite the objects. Both the arena and the objects were wiped with 40% EtOH after each session.

The rats were allowed to explore two identical objects for 4 min during the sample phase (C1 and C2). Twenty-four hr. later, the rats underwent a testing phase, during which they explored two copies of the sample objects (C3 and C4) for 4 min, but with one object moved to a corner location at the front of the box while the other maintained its original position. The amount of time each rat spent actively exploring the objects was recorded. A rat received credit for active exploration when its nose was directed within 2 cm of an object and either its head or vibrissae were moving, but not when it was standing on top of an object and looking away. Rats were required to explore objects for at least 15 s in both the sample and test phases to have reliable recognition memory. A discrimination ratio (DR) was calculated as the time the rat spent exploring the novel orientation subtracted by the time the rat spent exploring the familiar orientation during the first two min of the test phase and dividing by two. A positive discrimination ratio indicates intact OBL memory (Howland and Cazakoff, 2010; Howland et al., 2012).

Three days following object location testing, rats were tested in the object-in-place (OBIP) paradigm. During the sample phase, rats explored 4 different objects (D1, E1, F1, G1) located in the four corners of the arena for 5 min. Following a 1-h delay, rats were placed back in the arena for the test phase. During the test phase, rats again explored 4 identical copies of the objects (D2, E2, F2, G2) for 3 min, but in this case the position of 2 of the objects on the same side was switched (i.e., front object becomes the back object and vice-versa). Object-in-place memory is inferred when animals spend more time exploring the pair of objects that switched positions than the objects that remained in their original positions (Howland et al., 2012). Here a discrimination ratio was again calculated to determine the degree to which rats explored the novel objects. A positive discrimination ratio indicates intact OBIP memory.

### Histology

Rats were sacrificed 3 days after the OBIP memory task. Each rat was deeply anesthetized with 5% isoflurane and then transcardially perfused using physiological saline, followed by ice-cold 4% (w/v) formaldehyde fixative (pH = 7.4). The brains were extracted from the cranial vault and immersed in the same formaldehyde fixative for 48 h at 4°C. The brains were then sectioned in the coronal plane at 30 μm on a vibrating microtome (VT1200s, Leica Biosystems). Sections were collected and stored at -20°C in a cryoprotectant solution containing 30% (w/v) sucrose, 1% (w/v) polyvinylpyrrolidone, and 30% (v/v) ethylene glycol in 0.1 M PBS (pH = 7.4).

### Immunohistochemistry

We used a standard immunohistochemistry technique with commercially available antibodies for all of our experiments. Immunostaining was done on free-floating sections in six-well tissue culture plates under gentle agitation. To ensure consistent immunohistochemical processing, we processed all sections for each assay (i.e., DCX, reelin, GABAA β2/3receptorsubunit) in unison with treatment groups counterbalanced across all tissue plates. To confirm the specificity of our antibodies, we omitted the primary antibody from an additional well of free-floating sections. In the absence of the primary antibody, we were unable to detect any immunoreactive cells.

We used doublecortin (DCX) to label immature dentate granule cells, as previously described (Botterill et al., 2015). Brain sections underwent heat-induced epitope retrieval in sodium citrate buffer (pH = 6.0) at 85°C for 30 min. The sections were then blocked in 5% (v/v) normal goat serum (NGS), 1% (w/v) bovine serum albumin (BSA), and 0.5% (v/v) Triton X-100 in 0.1 M TBS (pH = 7.4), followed by incubation in a rabbit anti-DCX polyclonal primary antibody (1:1000, Cell Signaling Technology) diluted in blocking solution for 24 h. at room temperature. The next day, the sections were treated with 5% (v/v) H_2_O_2_ in 0.1 M TBS for 30 min to block endogenous peroxidase activity. The sections were then incubated for 1 h in biotinylated goat anti-rabbit secondary antibody (1:500, Vector Laboratories) diluted in 5% (v/v) NGS, 1% (w/v) BSA, and 0.5% (v/v) Triton X-100 in 0.1 M TBS, followed by avidin-biotin peroxidase complex (1:500, Vector Laboratories) for 1 h. Sections were then rinsed with 0.175 M sodium acetate (pH = 6.8) and visualized with 0.025% (w/v) DAB, 4.167% NiSO_4_, and 0.002% (v/v) H_2_O_2._

Reelin immunohistochemistry was conducted as previously described (Lussier et al., 2013a). Sections were treated with 0.3% H2O2 in 0.1 M PBS for 30 min to block endogenous peroxidase activity. The sections were then blocked in 5% normal horse serum (NHS), 1% (w/v) BSA, and 0.3% (v/v) Triton X-100 in 0.1 M PBS (pH = 7.4), followed by incubation in a mouse anti-reelin primary antibody (1:2000, Vector Labs) diluted in blocking solution for 48 h at room temperature. Following primary antibody, the sections were then incubated for 2 h. in biotinylated horse anti-mouse secondary antibody (1:500, Vector Labs) diluted in 5% (v/v) NHS, 1% (w/v) BSA, and 0.3% (v/v) Triton X-100 in 0.1 M PBS, followed by avidin-biotin peroxidase complex (1:500, Vector Laboratories) for 1 h. Sections were then rinsed with 0.175 M sodium acetate (pH = 6.8) and visualized with 0.033% (w/v) DAB and 0.007% (v/v) H_2_O_2._

GABAA β2/3 receptor subunit immunohistochemistry was conducted as follows. Sections first underwent heat-induced epitope retrieval in sodium citrate buffer (pH = 6.0) at 85°C for 30 min. Next, the sections were placed into a blocking solution containing 5% (v/v) NHS, 1% (w/v) BSA, and 0.5% (v/v) Triton X-100 in 0.1 M TBS for 30 min, and were then exposed to a monoclonal mouse anti-GABA_A_ β2/3 receptor primary antibody (clone bd17, 1:1000, Millipore/Sigma) diluted in blocking solution for 48 h at 4°C. Following this, the sections were treated with 5% (v/v) H_2_O_2_ in 0.1 M TBS for 30 min to block endogenous peroxidase activity. Next, the sections were incubated for 2 h in biotinylated horse anti-mouse secondary antibody (1:500, Vector Laboratories) diluted in 5% (v/v) NHS, 1% (w/v) BSA, and 0.5% (v/v) Triton X-100 in 0.1 M TBS, followed by avidin-biotin peroxidase complex (1:500, Vector Laboratories) for 1 h. The sections were then rinsed with 0.175 M sodium acetate (pH = 6.8) and visualized with 4.167% NiSO_4_ and 0.05% (w/v) glucose oxidase DAB_._

At the end of each immunohistochemical run, all sections were mounted onto glass slides using 0.2 M PB (pH = 7.4), air dried overnight, dehydrated using a series of graded alcohols, cleared in xylene, and cover slipped with Permount mounting medium (Fisher Scientific).

### Cell Counting

Immunohistochemical results were quantified as previously described (Lussier et al., 2013a; Botterill et al., 2015). All analyses were conducted by researchers blind to the treatment conditions. Immunostained sections were examined using a Nikon Eclipse E800 microscope with a motorized stage and digital camera (MicroFire, Optronics) connected to a dedicated stereology computer. The dentate SGZ (defined as a two-cell width zone in between the inner granule cell layer and the hilus) and granule cell layer (GCL) were traced at 4 X magnification using a computerized stereology program for DCX-ir cells. However, only the SGZ was traced for reelin and GABA_A_ β2/3-ir cells (StereoInvestigator, MicroBrightfield, Williston, VT, United States). DCX, reelin, and GABA_A_ β2/3-ir cells were counted in both the ipsilateral and contralateral hemispheres at 40 x magnification. All counts utilized unbiased stereology using a modified optical fractionator method that excludes cells in focus at the uppermost focal plane to reduce oversampling (Lussier et al., 2013a). The total number of cells was estimated using the following formula: N_total_ = ΣQ^-^ × 1/ssf × A(x, y step) / a(frame) × t/h. ΣQ^-^ represents the number of counted cells, ssf is the section sampling fraction (1 in 12), A(x, y step) is the area associated with each x, y movement (10,000 μm^2^), a(frame) is the area of the counting frame (3600 μm^2^), t is the weighted average section thickness, and h is the height of the dissector (12 μm) (Lussier et al., 2013a; Botterill et al., 2015). To avoid counting sectioning artifacts, we used a guard zone of 2 μm.

### Characterization of Immature DCX-ir Neurons

We utilized a dendritic categorization method (Lussier et al., 2013a; Botterill et al., 2015) to determine whether CORT or etanercept altered the dendritic morphology of immature DCX-ir neurons. A meander scan method was used to randomly select 100 DCX-ir cells from each rat. A researcher blind to experimental conditions then assigned the cell to one of six categories of dendritic complexity based on both the presence and extent of apical dendrites (see **Figures 5C,D**). The proliferative stage encompassed categories one (no process) or two (one small process). Category three (medium process reaching the granule cell layer) and category four (process reaching the molecular layer) comprised the intermediate stage of development. Finally, category five (one major process extending into the molecular layer) and category six (defined dendritic tree with delicate dendritic branching in the granule cell layer) represented mature cell development. Data are presented as the percentage of DCX-ir cells in each of the six categories.

### Statistical Analyses

All statistical analyses were done using IBM’s Statistical Package for Social Sciences v24. We used separate one-way ANOVA’s to assess the statistical significance of group differences in each measure (i.e., body weight, FST behavior, OBL, OBIP, DCX, reelin, GABA_A_ β2/3), and significant main effects were followed by *post hoc* comparisons using Tukey’s HSD. The criterion for statistical significance was *p* < 0.05. All graphs depict the mean ± standard error of the mean.

## Results

### CORT Had Significant Effects on Body Weight

**FIGURE 2:**
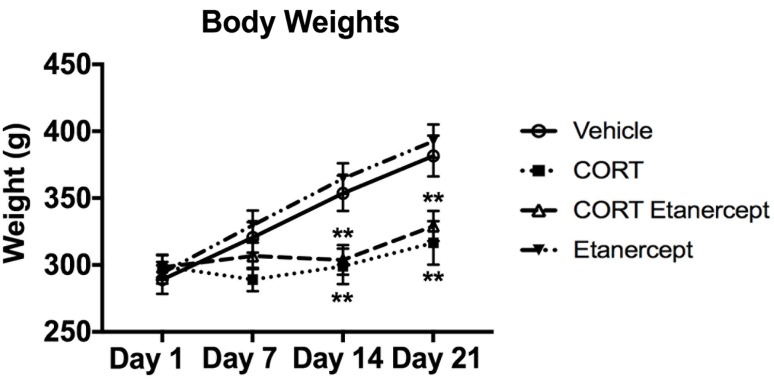
Effect of CORT and etanercept on body weight. The CORT rats weighed significantly less than the vehicle and etanercept rats on days 14 (*p* < 0.05) and days 21 (*p* < 0.05). In addition, the CORT + etanercept rats weighed significantly less than the vehicle or etanercept rats on days 14 (*p* < 0.05) and 21 (*p* < 0.05). All data are represented as means ± standard error of the mean.

**Figure 2** shows fluctuations in body weight in each group during the CORT injections. CORT had significant effects on body weight, consistent with previous findings from our lab (Lussier et al., 2013a,b). We found significant group differences on days 14 [*F*(3,23) = 7.414, *p* < 0.01] and 21 [*F*(3,23) = 7.295, *p* < 0.01]. *Post hoc* analyses revealed that the CORT-treated rats weighed significantly less than the vehicle and etanercept rats on day 14 (*p-*values < 0.05) and day 21 (*p*-values < 0.05). We also found that the CORT + etanercept rats weighed significantly less than the vehicle and etanercept rats on day 14 (*p*-values < 0.05) and significantly less than the etanercept rats on day 21 (*p* = 0.021).

### Etanercept Normalizes CORT-Induced Changes in Behavior on the FST

**FIGURE 3:**
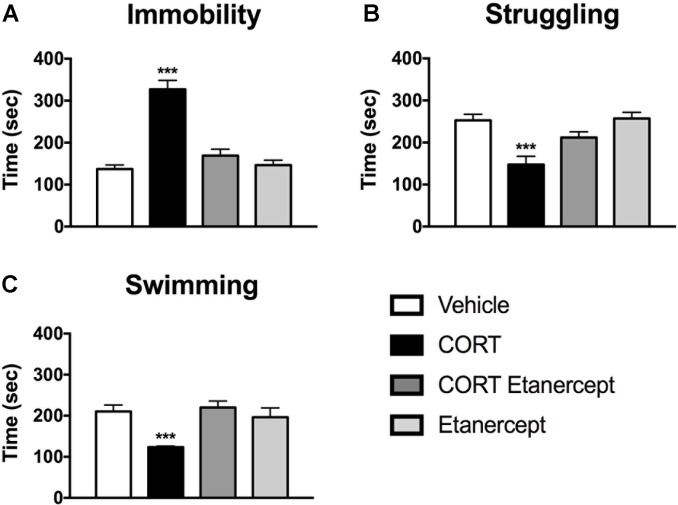
Effect of CORT and etanercept on FST behavior. **(A)** Shows the effect of treatment on time spent immobile. The CORT rats spent significantly more time immobile than did the vehicle, CORT + etanercept, and etanercept rats (*p* < 0.05). **(B)** Shows the effect of treatment on time spent struggling. The CORT rats spent significantly less time struggling compared to the vehicle, CORT + etanercept, and etanercept rats (*p* < 0.05). **(C)** Shows the effect of treatment on time spent swimming. The CORT rats spent significantly less time swimming than did the vehicle, CORT + etanercept, and etanercept rats (*p* < 0.05). All data are represented as means ± standard error of the mean.

**Figure 3** shows the results of the FST in each group. We found significant group differences in immobility [*F*(3,23) = 34.96, *p* < 0.001], struggling [*F*(3,23) = 10.39, *p* < 0.001], and swimming [*F*(3,23) = 7.57, *p* < 0.001] during the FST. *Post hoc* analyses indicated that the CORT rats spent significantly more time immobile, less time struggling, and less time swimming than the rats in all other groups (*p* values < 0.05). No other group differences were significant.

### Etanercept Normalizes CORT-Induced Deficits in OBL and OPIP Memory

**FIGURE 4:**
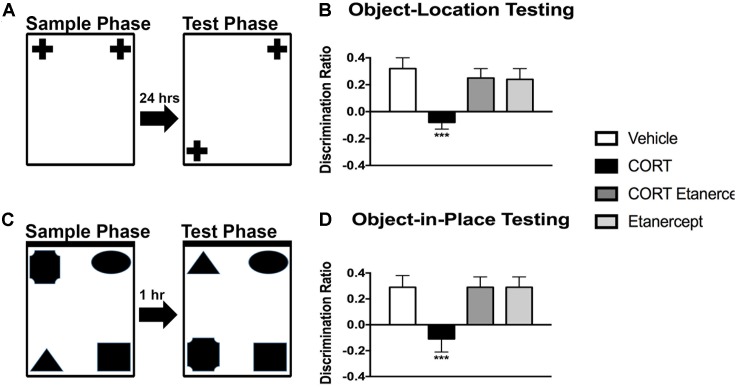
Effect of CORT and etanercept on cognition. **(A)** Shows a schematic of the object location memory test. **(B)** Shows the discrimination ratio in this test for the rats in each group. The CORT rats had a significantly lower discrimination ratio than did the vehicle, CORT + etanercept, and etanercept rats (*p* < 0.05). **(C)** Shows a schematic of the object-in-place memory test. **(D)** Shows the discrimination ratio in this test displayed by the rats in each group. The CORT rats had a significantly lower discrimination ratio than did the vehicle, CORT + etanercept, and etanercept rats (*p* < 0.05). All data are represented as means ± standard error of the mean.

**Figure 4** shows the results of the OBL and OBIP testing in each of the groups. We found significant group differences in both the OBL [*F*(3,23) = 5.858, *p* < 0.01] and OBIP [*F*(3,23) = 5.088, *p* < 0.01] memory tests. *Post hoc* analyses of these main effects revealed that the CORT rats had a lower discrimination ratio than all other groups in the OBL test (*p-*values < 0.05) and OBIP test (*p*-values < 0.05). No other group differences were significant.

### Etanercept Restores CORT-Induced Reductions in Hippocampal Neurogenesis

**FIGURE 5:**
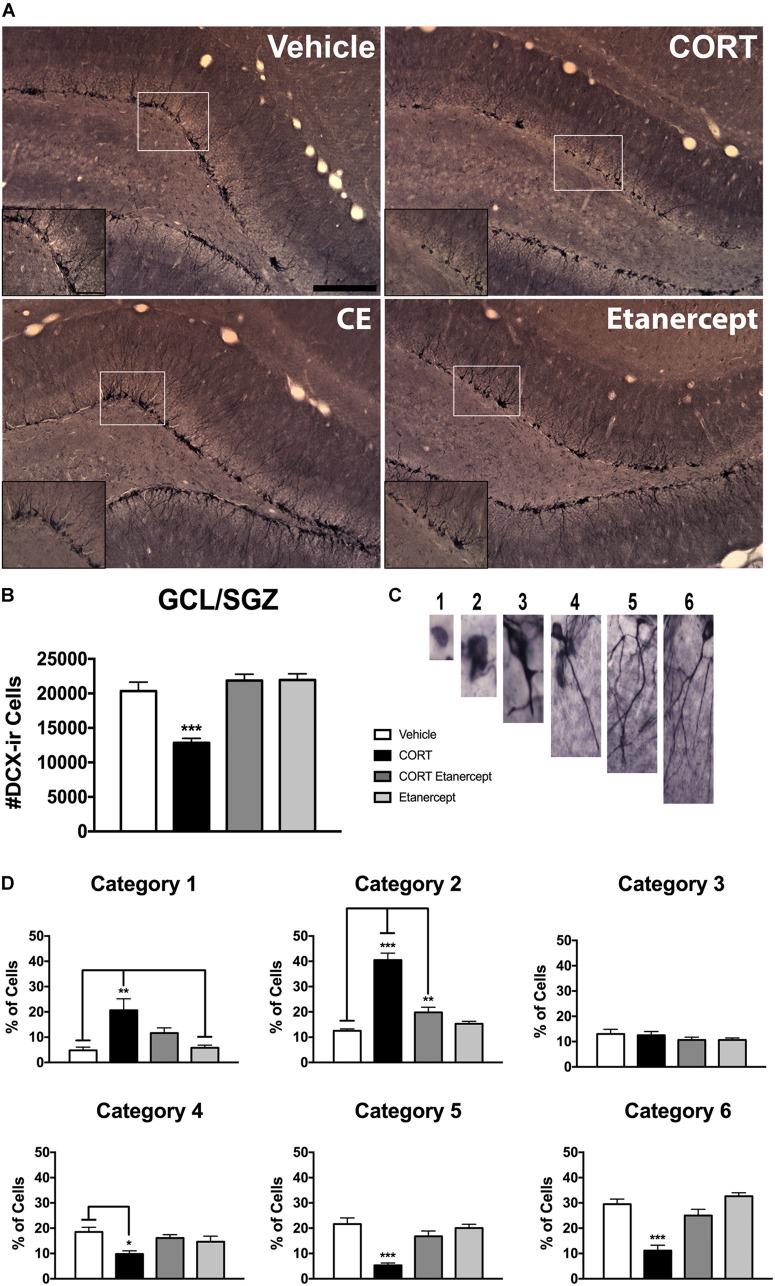
Effect of CORT and etanercept on DCX-ir and dendritic complexity. CORT treatment significantly impaired hippocampal neurogenesis, and etanercept treatment restored this. **(A)** Shows representitive photomicrographs of DCX expression in the dentate gyrus (scale bar = 200 μm). A higher magnification image is pictured in the insets (Scale bar = 50 μm). **(B)** Shows the effect of treatment on the number of DCx-ir cells in the subgranular zone. CORT-treated rats had significantly fewer DCX-ir cells than did the vehicle, CORT + etanercept, and etanercept rats (*p* < 0.05). **(C)** Shows representative photomicrographs of the six categories of dendritic complexity. **(D)** Shows the quantified categorization of dendritic complexity after analysis of a subset of DCX-ir cells. In general, the CORT rats had a higher percentage of cells in category 1 and category 2 and lower percentage of cells in categories 5 and 6, demonstrating that DCX-ir neurons are less well developed after CORT treatment. All data are represented as means ± standard error of the mean.

**Figure 5** shows the results of our DCX analyses. **Figure 5A** provides photomicrographs of DCX immunoreactivity in each of the four groups and **Figures 5B,D** show quantified differences among the groups. Consistent with previous results from our lab (Lussier et al., 2013a; Fenton et al., 2015), we found a significant main effect of group on the total number of DCX-ir cells [*F*(3,23) = 20.180, *p* < 0.001]. *Post hoc* analyses of this main effect revealed that the CORT rats had significantly fewer DCX-ir neurons compared to the vehicle, CORT-etanercept, and etanercept rats (*p*-values < 0.001). No other group differences were significant.

In relation to dendritic complexity, we also found a number of significant group differences. There were significant group differences in the % of neurons in category 1 [*F*(3,23) = 7.760, *p* < 0.01], category 2 [*F*(3,23) = 49.504, *p* < 0.001], category 4 [*F*(3,23) = 4.673, *p* < 0.05], category 5 [*F*(3,23) = 16.329, *p* < 0.001], and category 6 [*F*(3,23) = 21.852, *p* < 0.001]. *Post hoc* analyses showed that the CORT rats had a greater percentage of category 1 cells than did the vehicle (*p* = 0.002) and etanercept rats (*p* = 0.003). The CORT rats also had a greater % of category 2 cells than all other groups (*p*-values < 0.001), while CORT etanercept animals had a greater % of category 2 cells than vehicle rats (*p* = 0.042). CORT rats had a smaller % of category 4 cells than the vehicle rats (*p* = 0.009), and a smaller % of category 5 and category 6 cells than all other groups (*p-*values < 0.001). In summary, the CORT rats had more DCX-ir cells with less complex dendrites but the CORT + etanercept rats did not differ from vehicle rats in this respect.

### Etanercept Restores CORT-Induced Reduction in Hippocampal Reelin-ir Cells

**FIGURE 6:**
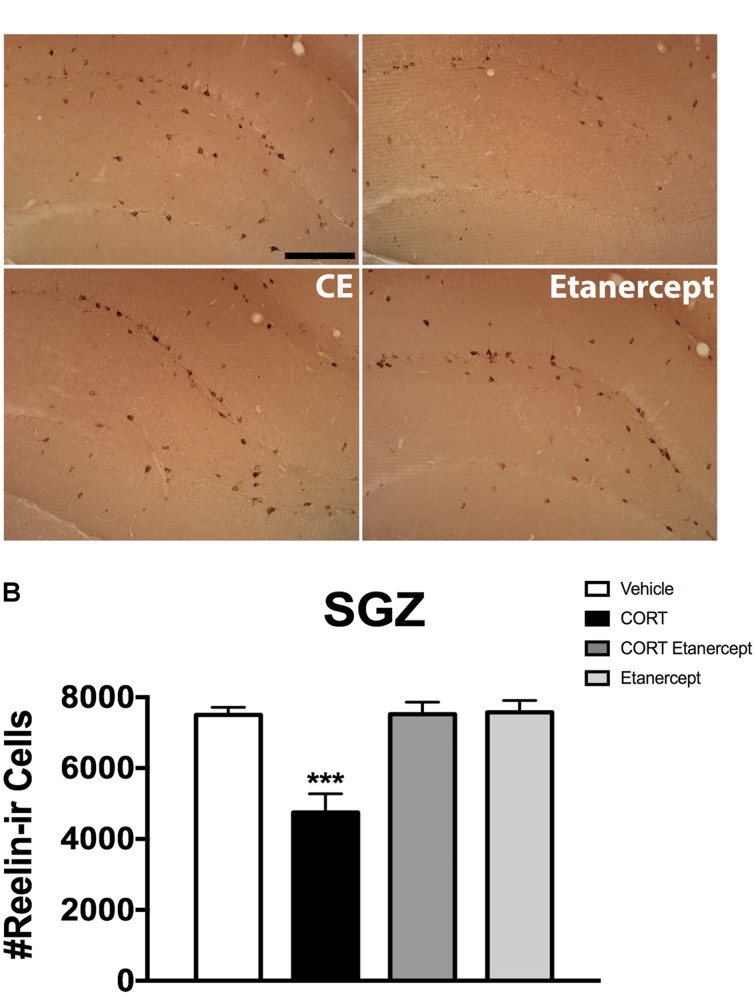
Effect of CORT and etanercept on the number of reelin-ir neurons. **(A)** Shows representative photomicographs of reelin-ir neurons in the dentate gyrus (scale bar = 200 μm). We focused our attention on reelin-ir cells in the proliferative subgranular zone. **(B)** Shows quantified values of reelin-ir neurons. The CORT rats had significantly fewer reelin-ir cells compared to the vehicle, CORT + etanercept, and etanercept rats (*p* < 0.05). All data are represented as means ± standard error of the mean.

**Figure 6** shows the results of the reelin immunohistochemistry. **Figure 6A** shows the distribution of reelin-ir neurons in the SGZ across the groups and **Figure 6B** shows the actual reelin-ir cell counts. Our stereological analyses revealed a significant effect of group on the number of reelin-ir cells in the SGZ [*F*(3,23) = 14.256, *p* < 0.001]. *Post hoc* analyses further revealed that the CORT rats had significantly fewer reelin-ir cells than the rats in all other groups (*p*-values < 0.001). No other group differences were significant.

### Etanercept Restores CORT-Induced Reduction in GABA_A_ β2/3-ir Cells in the SGZ

**FIGURE 7:**
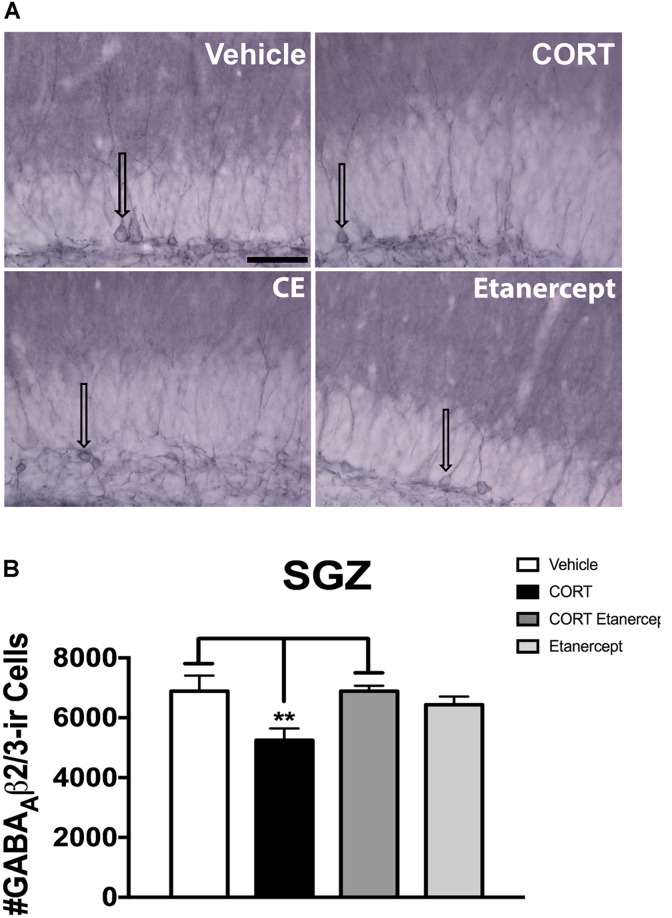
Effect of CORT and etanercept on the number of GABA_A_ β2/3-ir cells. **(A)** Shows representative photomicrographs of GABA_A_ β2/3 expression along the subgranular zone (scale bar = 200 μm). Arrows point to cell bodies showing GABA_A_ β2/3-ir. Note that in the vehicle rats, GABA_A_ β2/3 immunostaining appears to be concentrated on the plasma membrane of the cell bodies, whereas after CORT treatment the labeling is more diffuse and can be observed along the cytoplasm indicating receptor internalization. Etanercept partially rescues this effect. **(B)** Shows the quantified number of GABA_A_ β2/3-ir cells in each group. The CORT rats had significantly fewer GABA_A_ β2/3-ir cells compared to the vehicle and CORT + etanercept rats (*p* < 0.05). All data are represented as means ± standard error of the mean.

**Figure 7** shows the results of the GABA_A_ β2/3 immunohistochemistry. **Figure 7A** provides photomicrographs of GABA_A_ β2/3 immunoreactivity in each group and **Figure 7B** provides the results of our stereological cell counts. We found a significant main effect of group on the number of GABA_A_ β2/3ir cells in the SGZ [*F*(3,23) = 4.530, *p* < 0.05]. *Post hoc* analyses revealed that the CORT rats had significantly fewer GABA_A_ β2/3-ir cells than the vehicle rats (*p* = 0.022) and CORT + etanercept rats (*p* = 0.022), but not the etanercept only rats (*p* = 0.129). CORT induces internalization of GABA_A_ receptors in the SGZ (see arrows in **Figure 7A**) and this effect was also partially rescued by etanercept.

## Discussion

The results of this experiment show that prolonged exposure to the stress hormone CORT produces a cluster of effects characterized by increased immobility in the FST, impaired spatial memory on the OBL and OBIP tests, reduced hippocampal neurogenesis, fewer SGZ reelin-ir cells, and dampened GABA_A_ β2/3 expression levels. Importantly, semi-weekly etanercept given along with CORT prevented all these effects, suggesting that anti-TNF-α drugs could exert their antidepressant effects by facilitating key aspects of hippocampal plasticity.

The current experimental paradigm employed the use of individual housing along with CORT injections in adult rats, a protocol that we and others have extensively used in the past to analyze depressive-like behavior (see Sterner and Kalynchuk, 2010, for a review; Gregus et al., 2005; Johnson et al., 2006; Lussier et al., 2009, 2013a,b; Brummelte and Galea, 2010a,b; Fenton et al., 2015). While it has been noted that social isolation can increase depressive-like behavior in rodents and disrupt the neuroendocrine profile, during postnatal development and weaning period (Lapiz et al., 2003; Leng et al., 2004; Weiss et al., 2004; Colianna et al., 2013; Shetty and Sadananda, 2017a,b; Wang et al., 2017), behavioral alterations related to social isolation in adult rats have been reported only after longer isolation periods than those used in the present study (see Murinova et al., 2017). Furthermore, group housing adult rats can lead to increased cortisol levels, indicative of an increased stress response (Laber et al., 2008; Kamakura et al., 2016).

Prolonged exposure to CORT in rodents is known to produce a clear behavioral phenotype of depression, characterized by increased immobility in a FST and tail suspension test, decreased preference for sucrose, and decreased interest in sexual behavior (Marks et al., 2009; Sterner and Kalynchuk, 2010; Lussier et al., 2013a; Fenton et al., 2015). Our FST results are consistent with these previous findings, in that the CORT rats displayed increased immobility and decreased swimming and struggling compared to control rats. Importantly, etanercept reversed the effects of CORT on FST behavior. The CORT + etanercept displayed significantly less immobility than the CORT rats and in fact, they looked very much like control rats in this test. This was expected, because there have been previous studies that assessed the antidepressant effects of etanercept in the FST. For example, Krugel et al. (2013) reported that intraperitoneal injections of etanercept can reverse the effects of chronic restraint stress on FST immobility. Adding translational validity to the preclinical observations that TNF-α blockade has antidepressant effects, Raison et al. (2013) observed antidepressant effects of infliximab (a TNF-α inhibitor) in a clinical sample. Specifically, their work demonstrated that infusion of infliximab reduced scores on the Hamilton scale in patients with a high baseline level of inflammation. Furthermore, a recent systematic review and meta-analysis of seven randomized clinical trials demonstrated the antidepressant effectivity of several anti-TNF-α drugs (i.e., adalimumab, etanercept, infliximab, and tocilizumab) (Kappelmann et al., 2016). Therefore, our findings and the literature presented here suggest that TNF-α –induced inflammation is critically involved in the pathogenesis of depression, and therapies targeting this inflammation can produce therapeutic benefits. However, the mechanisms by which TNF-α antagonism can produce antidepressant effects are completely unknown.

Chronic stress is known to impair aspects of cognition, particularly hippocampal-mediated spatial memory. For example, deficits in Morris Water maze performance (Warner et al., 2013), fear conditioning (Hoffman et al., 2014), and OBL memory (Hattiangady et al., 2014) have all been observed after exposure to chronic stress. Inflammation can also impair performance on hippocampal-dependent forms of memory. This has been seen through studies using lipopolysaccharide, where impairments on context-object location discrimination were associated with high levels of proinflammatory cytokines, such as TNF-α (Czerniawski et al., 2015). Chronic restraint stress can also elevate hippocampal TNF-α levels, and this has been linked to impairments in a passive avoidance learning test (Azadbakht et al., 2015). Furthermore, studies of breast-cancer survivors reveal reduced hippocampal volumes and deficits in verbal memory performance that are associated with elevated circulating levels of TNF-α (Kesler et al., 2013). Here, in line with previous research, we report that CORT impairs both OBL and OBIP memory, and that etanercept restores memory performance to control levels. The CORT rats were not able to discriminate the novel object location in either test, whereas rats in all other groups, including the CORT + etanercept rats, performed normally. These findings reinforce the idea that TNF-α plays an important role in hippocampal-dependent memory because the OBL is a hippocampal dependent task, but interestingly, OBIP relies not only on the hippocampus, but also on functional connections between the hippocampus, perirhinal cortex, and medial prefrontal cortex (Barker and Warburton, 2015). These connections require intact NMDA receptor synaptic plasticity (Barker and Warburton, 2008) and it is notable that one action of hippocampal reelin (also restored by etanercept in this experiment) is to regulate the composition and trafficking of NMDA receptor subunits (Bosch et al., 2016b). Therefore, the restoration of reelin –ir cells by etanercept in this experiment could have played a role in the normalization of cognitive behavior.

One should consider that etanercept is a large molecule that is unable to cross the blood-brain barrier (Boado et al., 2010). Peripheral etanercept seems to be able to indirectly reduce CNS inflammation as a consequence of decreased peripheral inflammatory signals (Kerfoot et al., 2006). A particularly novel finding from this experiment was that peripheral administration of etanercept restored CORT-induced deficits in hippocampal neurogenesis. Reductions in hippocampal neurogenesis are a well-documented finding in the preclinical stress literature (Sliwowska et al., 2010; Lussier et al., 2013a; Fenton et al., 2015; Levone et al., 2015). However, the role of hippocampal neurogenesis in depression is a contentious issue, as ablation of hippocampal neurogenesis does not always induce depression-like behavior (Bessa et al., 2009), and antidepressant effects can be achieved without increases in neurogenesis (Hanson et al., 2011). Furthermore, studies of post-mortem tissue from depressed patients previously failed to find reductions in hippocampal neurogenesis (Reif et al., 2006); although more recent papers seem to refute that (Boldrini et al., 2012). Interestingly, Hill et al. (2015) convincingly demonstrated that increasing hippocampal neurogenesis through a transgenic mouse line reversed a CORT-induced depressive phenotype, suggesting that neurogenesis *per se* does play a contributing role in antidepressant responses. Moreover, when considering neurogenesis, one should focus not only on the number of newborn neurons, but also on the maturation of those neurons, including the sprouting of dendritic arborization and integration of new neurons into mature circuits that seem to be dampened in depression and rescued by effective antidepressant treatment (Mateus-Pinheiro et al., 2013a,b; Alves et al., 2017; Morais et al., 2017). Importantly, our data indicate that peripheral etanercept administration works in rescuing not only the number of new neurons but also the maturation rate of hippocampal DCX-ir cells, and this could be one mechanism by which etanercept exerts an antidepressant action.

How might peripheral administration of etanercept be acting to normalize CORT-induced deficits in neurogenesis? To our knowledge, this is the first study to report such an effect. It has been shown that peripherally produced TNF-α acts on toll-like receptor 4 on circumventricular organs and choroid plexus, which in turn stimulates microglia to secrete of pro-inflammatory cytokines (among them TNF- α), resulting in neurodegenerative events and an increase of apoptotic neuronal death (Bortolato et al., 2015). On the other hand, low levels of TNF-α are known to produce the opposite effect, promoting hippocampal neurogenesis through increased proliferation of neural precursor cells (Fan et al., 2011). Therefore, peripheral administration of etanercept likely decreases the expression of peripheral pro-inflammatory cytokines, which indirectly protects against the deleterious effects of CORT. This idea should be further examined in future studies.

Another novel finding from this experiment is that peripheral injections of etanercept normalize CORT-induced deficits in hippocampal reelin expression. Previous work in our lab has shown that CORT decreases the number of reelin-positive cells in the hippocampus (Lussier et al., 2009, 2013a; Fenton et al., 2015). We believe that the behavioral and neurobiological effects of etanercept observed here are secondary to an increase in hippocampal reelin expression: this idea is supported by our previous observation that heterozygous reeler mice, which express 50% of the normal levels of reelin, are more susceptible to the depressogenic effects of CORT (Lussier et al., 2011), by previous findings showing that the time course for the development of depressive-behavior in rats coincides with the time course for decreased reelin expression (Lussier et al., 2013a), that the tricyclic antidepressant imipramine rescues hippocampal reelin levels in a dose-dependent manner (Fenton et al., 2015), and that oxidative events may be operative in CORT-induced deficits on reelin-ir cells in the dentate subgranular zone (Romay-Tallon et al., 2010, 2015). This leads to the idea that blocking peripheral TNF-α through the administration of etanercept will decrease excitotoxic insults affecting reelin-ir cells in the dentate SGZ, thus restoring the normal release of reelin alongside proliferating neurons in the SGZ, which will facilitate the maturation and integration of immature neurons and rescue the behavioral phenotype (for a more detailed explanation of this hypothesis see Caruncho et al., 2016). Direct support for this idea comes from observations that reelin overexpressing mice have increased neurogenesis (Pujadas et al., 2010) and that exogenous reelin can recover cognitive deficits in mouse models of both Angelman syndrome and Alzheimers Disease (Pujadas et al., 2014; Hethorn et al., 2015). This also dovetails nicely with our finding of that etanercept restores both OBL and OBIP memory, as discussed above.

Finally, we have previously shown that repeated CORT injections reduce GABA_A_ receptor expression in the hippocampus (Lussier et al., 2013b). Here, we found that CORT decreased the number of GABA_A_ β2/3-ir cells within the subgranular zone, and induced GABA_A_ receptor internalization, and etanercept restored this to control levels. TNF-α alters the surface expression of AMPA and GABA_A_ receptors by increasing AMPA receptor expression and decreasing GABA_A_ receptors (Stellwagen et al., 2005), which may contribute to the excitotoxic effects of upregulated TNF-α in a chronic stress paradigm (Bortolato et al., 2015). The normalization of GABA_A_ receptor expression in SGZ cells by peripheral etanercept may represent a neuroprotective mechanism that is necessary to facilitate SGZ plasticity and restore hippocampal neurogenesis. Indeed, much like reelin, GABA released from local interneurons exerts a depolarizing effect on immature neurons during the time when they express DCX, promoting neuronal differentiation and maturation (Song et al., 2013). In the absence of GABA, neuronal survival is jeopardized. Therefore, the influence of etanercept on GABA function is likely quite important for its antidepressant effects.

It should also be considered that etanercept is also affecting other body organs: For example, clinical studies in rheumatoid arthritis have indicated that etanercept -prescribed because of its anti-inflammatory activity-, can increase the risk of developing inflammatory bowel disease, lung infections, and pancytopenia (Thavaranjah et al., 2009; Santana et al., 2012; O’Toole et al., 2016), but these side effects of etanercept are not frequently reported and the medication is generally well tolerated.

## Conclusion

We have shown that peripheral injections of the TNF-α inhibitor etanercept normalize the depressogenic effects of repeated CORT administration, possibly through a restoration of hippocampal reelin expression, hippocampal neurogenesis, and GABA_A_ β2/3-ir cells in the dentate subgranular zone. These results add to the body of literature suggesting that inflammatory events are a critical component of the pathogenesis of depression and suggest that reelin may be a key link in the relationship between the immune system and brain. Future work should examine whether blocking reelin or its signaling mechanisms can abolish the antidepressant effect of etanercept.

## Author Contributions

All authors contributed to the design of the experiment and interpretation of the data. KB and EF acquired and analyzed the data. KB wrote the first draft and developed the figures and LK and HC edited and finalized the manuscript.

## Conflict of Interest Statement

The authors declare that the research was conducted in the absence of any commercial or financial relationships that could be construed as a potential conflict of interest.
